# Spontaneous Rupture of Urinary Bladder: Two Case Reports and Review of Literature

**DOI:** 10.3389/fsurg.2021.721705

**Published:** 2021-11-02

**Authors:** Yue Zhang, Shuo Yuan, Rami W. A. Alshayyah, Wankai Liu, Yang Yu, Chen Shen, Hang Lv, Lijie Wen, Yi He, Bo Yang

**Affiliations:** Department of Urology, The Second Hospital of Dalian Medical University, Dalian, China

**Keywords:** bladder rupture, spontaneous, diagnosis, treatment, systematic reviews

## Abstract

**Objectives:** Spontaneous rupture of the urinary bladder (SRUB) is extremely rare and might be misdiagnosed, leading to a high mortality rate. The current study aimed to identify the cause, clinical features, and diagnosis strategy of SRUB.

**Methodology:** We presented a case report for two women (79 and 63 years old) misdiagnosed with acute abdomen and acute kidney injury, respectively, who were finally confirmed to have SRUB by a series of investigations and exploratory surgery. Meanwhile, literature from multiple databases was reviewed. PubMed, the Chinese National Knowledge Infrastructure (CNKI), the Chinese Biological Medical Literature Database (CBM), WANFANG DATA, and the Chongqing VIP database for Chinese Technical Periodicals (VIP) were searched with the keywords “spontaneous bladder rupture” or “spontaneous rupture of bladder” or “spontaneous rupture of urinary bladder.” All statistical analyses were conducted using SPSS 20.0 software.

**Results:** A total of 137 Chinese and 182 English literature papers were included in this article review. A total of 713 SRUB patients were analyzed, including the two patients reported by us. The most common cause of SRUB was alcohol intoxication, lower urinary tract obstruction, bladder tumor or inflammation, pregnancy-related causes, bladder dysfunction, pelvic radiotherapy, and history of bladder surgery or bladder diverticulum. Most cases were diagnosed by exploratory laparotomy and CT cystography. Patients with extraperitoneal rupture could present with abdominal pain, abdominal distention, dysuria, oliguria or anuria, and fever. While the main symptoms of intraperitoneal rupture patients could be various and non-specific. The common misdiagnoses include acute abdomen, inflammatory digestive disease, bladder tumor or inflammation, and renal failure. Most of the patients (84.57%) were treated by open surgical repair, and most of them were intraperitoneal rupture patients. Overall, 1.12% of patients were treated by laparoscopic surgery, and all of them were intraperitoneal rupture patients. Besides, 17 intraperitoneal rupture patients and 6 extraperitoneal rupture patients were treated by indwelling catheterization and antibiotic therapy. Nine patients died of delayed diagnosis and treatment.

**Conclusions:** SRUB often presents with various and non-specific symptoms, which results in misdiagnosis or delayed treatment. Medical staff noticing abdominal pain suggestive of peritonitis with urinary symptoms should be suspicious of bladder rupture, especially in patients with a history of bladder disease. CT cystography can be the best preoperative non-invasive examination tool for both diagnosis and evaluation. Conservative management in the form of urine drainage and antibiotic therapy can be used in patients without severe infection, bleeding, or major injury. Otherwise, surgical treatment is recommended. Early diagnosis and management of SRUB are crucial for an uneventful recovery.

## Introduction

Spontaneous rupture of the urinary bladder (SRUB) is rare and is often a life-threatening condition usually caused by abdominal or pelvic trauma (1). Unfortunately, the diagnosis and treatment of SRUB are often delayed and missed; most cases are discovered during laparotomy. Surgical management is the recommended treatment for SRUB (2). However, the literature proved that conservative treatment by indwelling catheterization could also achieve good results for specific cases (3). Only a small case series of SRUB were found in the literature. Herein, we report two cases and review the literature to illustrate the clinical features, diagnosis, and treatment of SRUB.

## Case Presentation

### Case 1

**Figure 1:**
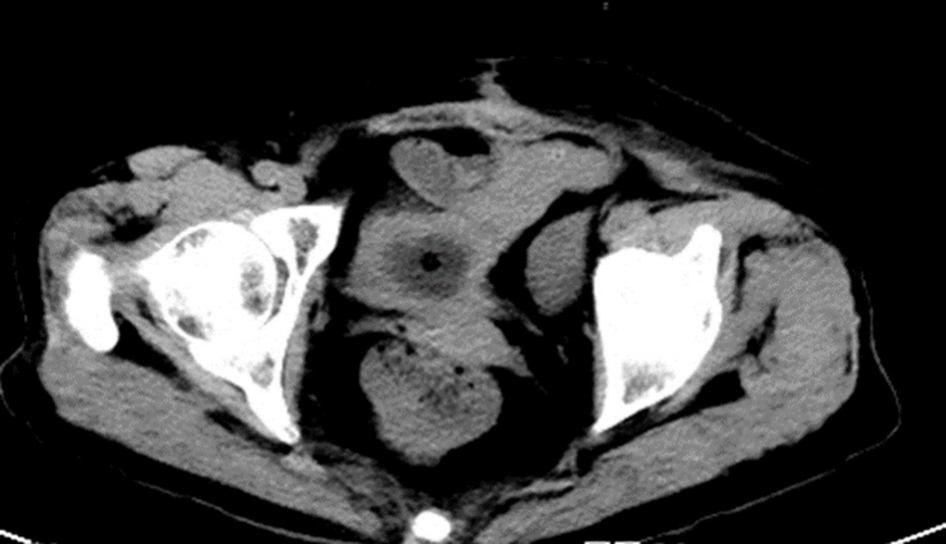
Coronal section CT scan of case 1. The CT scan demonstrated an indwelling catheter in the bladder and a small amount of fluid collection in the abdominopelvic cavity.

A 79-year-old woman with a history of transurethral resection of bladder tumor (TURBT) 2 years ago presented to the emergency department with sudden diffuse abdominal pain acute urinary retention of 24 h. The patient denied any history of trauma. Upon arrival at our hospital, vital signs were as follows: blood pressure, 117/65 mmHg; heart rate, 130/min; respiration rate, 28/min; and body temperature, 36.8°C. Physical examination revealed a distended abdomen with diffuse suprapubic tenderness. Laboratory investigations showed an elevated white blood cell (WBC) count (11.96 × 10^9^/L), serum creatinine (420 μmol/L), and urea (5.4 mmol/L). A Foley catheter was inserted and drained 250 mL of blood-stained urine. A CT scan (Figure 1) demonstrated both blood clots in the bladder and a small amount of fluid collection in the abdominopelvic cavity with a suspicious bladder wall defect in the bladder dome. Cystography showed intra-peritoneal contrast leakage and confirmed a defect at the dome of the urinary bladder. The patient's symptoms strongly suggested peritonitis from a ruptured bladder. As a result, emergency physicians and the urologist planned to repair the ruptured bladder by surgery. As the patient had a short course of disease and no history of intraperitoneal surgery, we chose laparoscopic bladder repair instead of laparotomy. An indwelling catheter was inserted for 14 days to allow the bladder defect to heal. Another cystogram was performed prior to catheter removal with no abnormal findings. The patient was discharged with a regular follow-up in the outpatient clinic.

### Case 2

**Figure 2:**
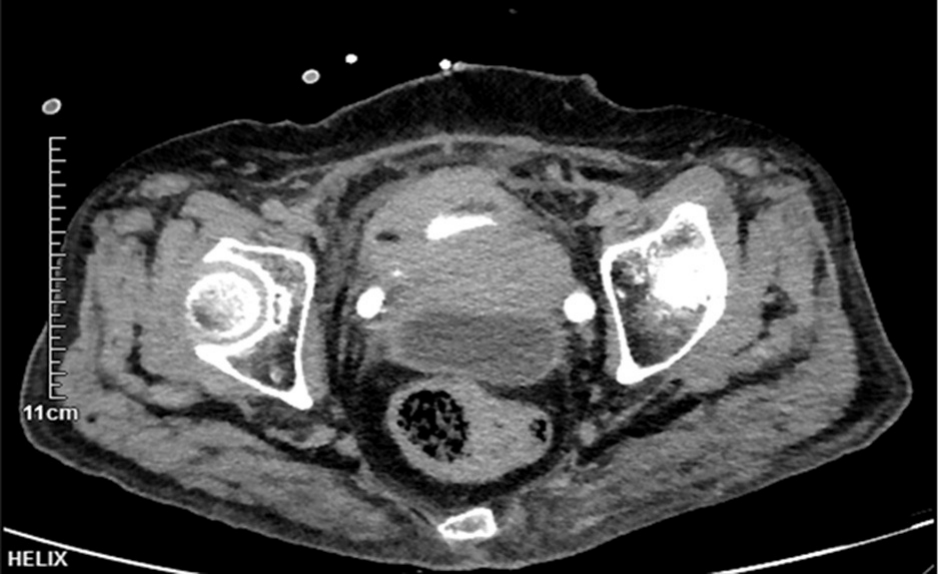
Coronal section CT urography of case 2. The bladder was filled with a contrast agent. The wall of the bladder was thickened. The CT scan demonstrated free fluid and thick-wall cysts in the pelvic cavity behind the uterus.

A 63-year-old woman with a history of diabetes, diabetic nephropathy, and neurogenic bladder visited the emergency department with complaints of recurrent lower abdominal pain and fever for 3 days. Vital signs were as follows: blood pressure, 120/70 mmHg; heart rate, 110/min; respiration rate, 25/min; and body temperature, 36.9°C. Physical examinations showed tenderness throughout the abdomen by palpation. Her laboratory investigations showed a white blood WBC count of 9.95 × 10^9^/L, serum albumin of 28.3 g/L, and creatinine of 424.6 μmol/L. A CT scan (Figure 2) demonstrated free fluid and a cyst-like mass between the bladder and uterus. The bladder water injection experiment was negative. And the drained fluid from the abdominal puncture demonstrated a creatinine level of 501.50 μmol/L, which was similar to the plasma creatinine level. Peritoneal puncture fluid examination showed that the Rivalta test was (+), the number of nucleated cells was 76,000/UL, and neutrophils were 90%. Escherichia coli was cultured in the peritoneal drainage fluid, the same as in a urine culture. The initial diagnosis was urinary tract infection, chronic renal failure, neurogenic bladder and pelvic effusion, and infection. The patient was treated with antibiotics, indwelling catheterization, and abdominal puncture drainage. The patient's level of serum creatinine gradually decreased to 194 μmol/L on the 7th day. However, there was low urine output and high peritoneal drainage. After multidisciplinary consultation and discussion, we suspected bladder rupture. A bladder rupture was confirmed by a methylene blue test and cystoscope that demonstrated a defect at the posterior lateral bladder wall. Laparotomy was performed, and a perivesical multilocular abscess was found during the operation. Multiple abscesses were drained, and repair of the bladder wall defect was performed in two layers. The patient made an uneventful recovery and was discharged after 14 days with a normal serum creatinine level and regular outpatient follow-up.

## Literature Review

### Search Strategy

A literature search was performed according to the Preferred Reporting Items for Systematic Reviews. The PubMed, CNKI, CBM, WANFANG DATA, and VIP databases were searched to identify reports on bladder rupture up to November 2020. The keywords used in our search strategy included: “spontaneous bladder rupture” OR “spontaneous rupture of bladder” OR “spontaneous rupture of urinary bladder.” There were no language restrictions. We focused on human studies. All selected articles were further manually searched to identify additional relevant articles.

### Inclusion Criteria and Exclusion Criteria

Studies were included if patients were diagnosed with spontaneous rupture of the urinary bladder (SRUB), and the treatment plan and results were reported. We excluded the following studies: (1) repeated studies: In cases of multiple reporting from the same single author, only the most comprehensive study was included; (2) letters, editorials, clinical experience studies; (3) laboratory articles, neonates, children, animal, and nonhuman studies; and (4) literature that failed to offer a full text or abstract.

### Data Extraction

After a full-text assessment, two investigators extracted data from the eligible studies using a standardized template, and their choices were corroborated by a third reviewer. The main data extracted from the eligible studies included: first author, sample size, age, gender, underlying diseases, causes, symptoms, laboratory and imaging findings, preliminary diagnosis, treatment plan, and outcome. All the data were collected and analyzed using SPSS 20.0 software.

### Evidence Synthesis

**Figure 3:**
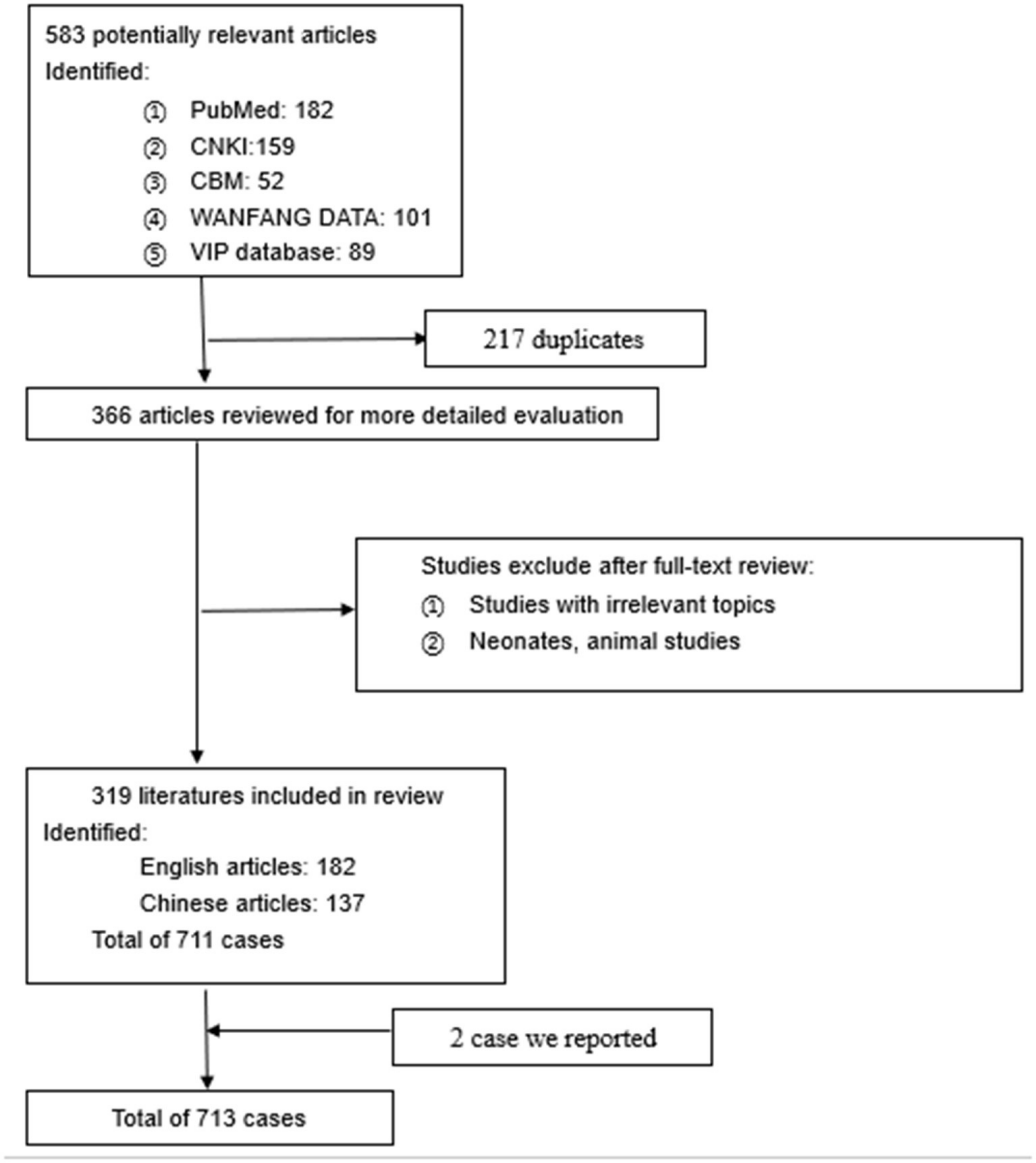
The process of the literature review.

Overall, we included 319 studies with 713 cases for qualitative analysis. Our initial search identified 583 articles. A total of 217 duplicated articles were excluded. Overall, 366 articles were selected for further review *via* full-text reading. After applying the selection criteria, we excluded three articles because they were animal cases, 36 articles that were infant cases, and eight articles that had irrelevant topics. We identified 319 articles with 713 patients for review. The process of the article search is shown in Figure 3. All studies were published between 1945 and 2020. The cases were conducted in the United States of America, China, Japan, England, France, and Germany. The extracted data from these 319 studies are outlined in Table 1. Most of the reported patients were men; ages ranged from 30 to 60 years.

**Table 1:** Basic characteristics of cases included.

	**Number**	**Ratio (%)**
**Gender** **(***n*** = 713)**		
Male	498	69.85
Female	202	28.33
Not mentioned	13	1.82
**Age** **(***n*** = 713)**		
<30	49	6.87
30–60	122	17.11
>60	66	9.26
Not mentioned	476	66.76
**Predisposing factors or underlying diseases** **(***n*** = 713)**		
**Pregnancy related (n** **=** **54)**		
Late pregnancy	21	7.57
Childbirth	24	
Postpartum urinary retention	9	
**Lower urinary tract obstruction** **(***n*** = 131)**		
Benign prostatic hyperplasia	98	18.37
Bladder or urethral calculi	16	
Urethral stricture	10	
Bladder outlet obstruction	5	
Pelvic organ prolapse	2	
**Bladder surgery or bladder diverticulum** **(***n*** = 24)**		
History of transurethral resection of bladder tumor	3	3.37
History of bladder repair/partial cystectomy	11	
History of renal transplantation	3	
History of bladder artery embolization	1	
Bladder diverticulum	6	
**Bladder dysfunction** **(***n*** = 42)**		
History of orthotopic ileocolon continent urinary reservoir	24	5.89
History of augmentation enterocystoplasty	5	
Neurogenic bladder	13	
**Bladder tumor or inflammation** **(***n*** = 91)**		
Bladder tumor	35	12.76
Chronic bladder inflammation	56	
**Pelvic disease invasion** **(***n*** = 3)**		
Colon cancer invasion	1	0.42
Pelvic inflammatory invasion	2	
**Neurological/psychiatric disease** **(***n*** = 10)**		
Disturbance of consciousness	8	1.4
Depression	2	
**Pelvic radiotherapy**	25	3.51
**Drunk**	280	39.27
**Strenuous activity**	4	0.56
**Long-term maintenance hemodialysis**	2	0.28
**Idiopathic**	47	6.59
**Mode of diagnosis** **(***n*** = 212)**		
Exploratory laparotomy	110	51.89
Cystography	48	22.64
Computed tomography	30	14.15
Cystoscope	17	8.02
Meilan test	7	3.3

## Result

The most common causes for SRUB were alcohol intoxication (39.27%), lower urinary tract obstruction (18.37%), bladder tumor or inflammation (12.76%), pregnancy-related causes (7.57%), bladder dysfunction (5.89%), pelvic radiotherapy (3.51%), history of bladder surgery or bladder diverticulum (3.37%), neurological or psychiatric diseases (1.4%), strenuous activity (0.56%), pelvic disease invasion (0.42%), long-term maintenance hemodialysis (0.28%), and idiopathic rupture (6.59%).

Most of the reported cases were diagnosed by exploratory laparotomy (51.89%), cystography (22.64%), computed tomography (14.15%), cystoscopy (8.02%), and Meilan tests (3.3%).

Patients with extraperitoneal rupture presented with abdominal pain, abdominal distention, dysuria, oliguria or anuria, and fever. While the main symptoms of intraperitoneal rupture patients could be various and non-specific. The patients' main symptoms are shown in Table 2.

**Table 2:** Main symptoms of cases included.

	**Intraperitoneal rupture**	**Extraperitoneal rupture**
	**(***n*** = 458)**	**(***n*** = 54)**
Abdominal pain	447	51
Abdominal distention	17	2
Dysuria	49	11
Oliguria or anuria	17	1
Hematuria	24	0
Fever	6	1
Nausea and vomiting	26	0
Diarrhea	1	0
Edema	1	0
Consciousness disorder	2	0

A total of 154 patients were reported to be misdiagnosed, as summarized in Table 3. The patients were misdiagnosed with acute abdomen, inflammatory digestive disease, bladder tumor or inflammation, and renal failure in a ratio of 78.57, 11.69, 5.84, and 4.55%, respectively.

**Table 3:** Misdiagnosis types of cases included.

	**Number**	**Ratio (%)**
**Acute abdomen** **(***n*** = 121)**		
Gastrointestinal perforation	27	78.57
Peritonitis	24	
Intestinal obstruction	20	
Appendicitis	11	
Mesenteric ischemia	3	
Volvulus	1	
Torsion of ovarian cyst	1	
Unknown	34	
**Inflammation of digestive system** **(***n*** = 18)**		
Pancreatitis	13	11.69
Gastroenteritis	3	
Pelvic infection	1	
Cirrhosis	1	
**Bladder tumor or inflammation** **(***n*** = 9)**		
Bladder tumor	5	5.84
Bladder infalmmation	4	
**Renal failure**	7	4.55

Overall, 84.57% of patients were treated by open surgery, and most of them were intraperitoneal rupture patients. In total, 1.12% of patients were treated by laparoscopic surgery, and all of them were intraperitoneal rupture patients. Besides, 17 intraperitoneal rupture patients and 6 extraperitoneal rupture patients were treated by indwelling catheterization and antibiotic therapy. Nine patients died due to delayed diagnosis and treatment. The treatment plan is summarized in Table 4. Usually, an indwelling catheterization duration lasted from 1 to 3 weeks.

**Table 4:** Treatment types of cases included.

	**Number**	**Ratio (%)**
**Open surgery** **(***n*** = 603)**		
Intraperitoneal rupture	364	84.57
Extraperitoneal rupture	39	
Not mentioned	200	
**Laparoscopic surgery** **(***n*** = 8)**		
Intraperitoneal rupture	8	1.12
**Catheterization** **(***n*** = 45)**		
Intraperitoneal rupture	17	6.31
Extraperitoneal rupture	6	
Not mentioned	22	
**Preoperative death** **(***n*** = 9)**		
Intraperitoneal rupture	1	1.26
Extraperitoneal rupture	1	
Not mentioned	7	
**Not mentioned**	48	6.73

## Discussion

Spontaneous rupture of the bladder is rare (<1%); the incidence is around 1 in every 126,000 people. The most common cause of bladder rupture is trauma (96%). And the bladder is most vulnerable when distended and can rupture from the weakest point. Extraperitoneal rupture of the bladder occurs in ~60–65% of cases, and intraperitoneal rupture in 25% (1). SRUB (intraperitoneal or extraperitoneal rupture) reported in the literature is a rare and life-threatening event (4). An accurate diagnosis followed by surgical intervention is the key for a successful outcome. Only 319 literature papers of 713 cases were included in our study; most of them were middle-aged men.

By analyzing the 713 cases, we concluded that the primary etiology of SRUB is the weakness of the bladder wall. Therefore, the bladder would rupture at the weak area during urination and increasing abdominal pressure (5). Alcohol intoxication is considered the most common predisposing factor to SRUB, can involve increased urine volume, causing bladder distention, decreasing perception of desire to void, and blunt trauma (6–8). Urinary bladder ruptures are attributed to a variety of predisposing factors, including but not limited to bladder surgery, such as TURBT, bladder repair or partial cystectomy, renal transplantation, bladder artery embolization, causing bladder wall weakness such as in bladder diverticulum (5), tumor infiltration, and inflammation which can also cause bladder local ulceration and necrosis (9, 10). Chronic urinary retention and recurrent urinary tract infections due to bladder dysfunction like neurogenic bladder also predispose a patient to bladder wall weakness and bladder rupture (11). Chronic urinary retention due to benign prostatic hyperplasia (BPH), bladder or urethral calculi, urethral stricture, female bladder outlet obstruction, and female pelvic organ prolapse can also lead to weakness of the bladder wall. Pelvic tumor radiotherapy is a possible cause of bladder complications, including inflammatory infiltrates, fibrosis, necrosis, and cellular atypia (12–14). Chronic urine retention during the prenatal period might also cause SRUB (15).

Other causes of excessive bladder filling can be responsible for rupture of the bladder.

Extraperitoneal bladder rupture most commonly presents with abdominal pain and dysuria. In contrast, intraperitoneal rupture has a classic triad consisting of macroscopic hematuria, abdominal pain, difficulty, or inability to void. Other presentations of bladder ruptures could be various and non-specific, ranging from anuria with free fluid to abdominal distention and oliguria. The diagnosis of SRUB is challenging; therefore, most patients were misdiagnosed with acute abdomen and inflammation of the digestive system.

Case 2 of our report was misdiagnosed as renal failure. This is because when urine comes in contact with the peritoneum, the higher concentrations of creatinine and urea nitrogen in the urine allows for concentration gradient diffusion, thus resulting in continuous reabsorption of creatinine, urea nitrogen, and potassium in the urine *via* the peritoneum into the systemic circulation. Therefore, the diagnosis will be delayed and missed for several days (5, 6, 16).

Early diagnosis and treatment of SRUB are closely linked to better prognosis (11, 12). Exploratory laparotomy is considered to be the gold standard of diagnosis as almost all the reported cases were diagnosed intraoperatively during a laparotomy for acute peritonitis. After literature review, we found that CT cystography can be the best recommended preoperative evaluation for suspected bladder rupture, and it can allow simultaneous assessment of multiple abdominal organs (17–19).

There are no specific guidelines for the treatment of SRUB. The European Association of Urology guidelines recommend that an intraperitoneal bladder rupture should always be managed by standard surgical repair because it might lead to a life-threatening condition due to the risk of abdominal sepsis and peritonitis. In contrast, conservative management can be considered for extraperitoneal bladder rupture (1, 4). However, after literature review, we found that conservative management proved to be a successful treatment for both intraperitoneal and extraperitoneal bladder rupture under particular situations (3) without severe infection, bleeding, or major injuries. The principles of conservative treatment are adequate urine drainage and antibiotic therapy. For urine drainage, an indwelling catheter or puncture drainage catheter may be used. Laparoscopic surgery can be performed for patients with definite intraperitoneal rupture without severe abdominal infection and other organ injury (5).

## Conclusion

SRUB often presents with non-specific symptoms, which results in missed or delayed diagnosis. When patients present with urinary symptoms and abdominal pain suggestive of peritonitis, the possibility of bladder rupture should be taken into consideration, especially those with a history of bladder disease. CT cystography is considered the best accurate non-invasive diagnostic and evaluation tool for suspected bladder rupture. Conservative management is recommended for patients without severe infection, bleeding, or major injuries. The principles of conservative treatment are adequate urine drainage and antibiotic therapy. Early diagnosis and management of SRUB are crucial for an uneventful recovery.

## Data Availability Statement

The original contributions presented in the study are included in the article/supplementary material, further inquiries can be directed to the corresponding author/s.

## Ethics Statement

The studies involving human participants were reviewed and approved by the Second Hospital of Dalian Medical University. The patients/participants provided their written informed consent to participate in this study. Written informed consent was obtained from the individual(s) for the publication of any potentially identifiable images or data included in this article.

## Author Contributions

BY, YY, and RA: protocol/project development. YZ, SY, WL, and YH: data collection and management. YZ, SY, WL, CS, and HL: data analysis. YZ, SY, and RA: manuscript writing and editing. All authors contributed to the article and approved the submitted version.

## Conflict of Interest

The authors declare that the research was conducted in the absence of any commercial or financial relationships that could be construed as a potential conflict of interest.

## Publisher's Note

All claims expressed in this article are solely those of the authors and do not necessarily represent those of their affiliated organizations, or those of the publisher, the editors and the reviewers. Any product that may be evaluated in this article, or claim that may be made by its manufacturer, is not guaranteed or endorsed by the publisher.
